# Analysis of the mechano-acoustic influence of the tympanic cavity in the auditory system

**DOI:** 10.1186/s12938-016-0149-2

**Published:** 2016-03-31

**Authors:** A. Garcia-Gonzalez, C. Castro-Egler, A. Gonzalez-Herrera

**Affiliations:** Department of Civil and Materials Engineering, University of Malaga, Calle Doctor Ortiz Ramos (Teatinos), 29071 Málaga, Spain; Calle Nuño Gómez, 20 2ºA, 29008 Málaga, Spain

**Keywords:** Ear canal, Middle ear, Tympanic membrane, Tympanic cavity, Finite element analysis, Resonance

## Abstract

**Background:**

The main objective of this paper is to study the mechanical influence of the tympanic cavity (TC) in the auditory system (AS). It is done for a frequency range from 0.1 to 20 kHz and the pressure source was applied in the external ear canal (EEC) entrance.

**Methods:**

Numerical simulations were developed for seven different models by means of finite element model. On the basis of an EEC finite elements model, the additional elements are coupled and removed in order to evaluate the contribution of the TC. Tympanic membrane, ossicular chain, simplified cochlea and TC were modeled and simulated in four different combinations.

**Results:**

Pressure, velocity, and displacement measures were obtained in AS key points in order to be compared with experimental results. Umbo and stapes transfer functions have been represented.

**Conclusions:**

The main conclusion is that we find evidence that the presence of the TC in the AS introduces a second resonance in middle ear transfer functions at frequencies above 3 kHz.

## Background

The mechanics of the outer and middle ear have been the objective of much research and many publications. There is a common position regarding the main behavior of the system, especially at lower frequencies, however at higher frequencies there are still some controversies. At lower frequencies (at the range below 1–2 kHz), the classic piston-like motion model is widely accepted to described the mechanics of the sound transmission. Experimental and numerical research confirms this basic model.

Nevertheless, at frequencies above this range, different coupled mechanisms appear which are difficult to identify. Aspects as how the tympanic cavity (TC) couples to the tympanic membrane (TM), the influence of the ossicular system on the dynamic of the membrane or the effect of the external ear canal (EEC) over the eardrum motion and transfer functions are certainly unclear nowadays.

There are some common surgical interventions, as tymplanoplasties or bone substitution by prosthesis, which solve hearing problem but sometimes derive on an audition quality lost. For the patient, it becomes manifest in limitation on the speech intelligibility or difficulties to appreciate music. This is normally due to changes on the transmission of higher frequency sounds. A better understanding of the sound transmission mechanisms at these frequencies is the base for proper interventions or therapies. For instance, in the case of a tympanoplasty, the change on the membrane thickness change its behavior at higher frequencies.

The main objective of this paper is to find evidence of the TC role in the transfer function. This has a double benefit, on the one hand we obtain a better comprehension of the behavior of the system, on the other hand we can shed light on the interpretation of experimental work which commonly has to manipulate the TC in order to carry out the experiment.

There is a great deal of work published about the influence of the ear canal (EC), TM and ossicular chain (OC) in the auditory system (AS). Some papers show experimental measurements varying TC conditions in order to deduce the TC role in the AS. Otherwise, experimental difficulties limit their finding and it is not clear how the TC affects TM motion at high frequencies.

Regarding numerical model and particularly in finite element method (FEM), only a few papers have included TC in their Models [1–6], and only some of them [1–3] focused on the specific role that the EC, TM, and TC connection plays in the human AS. There is previous FEM work on TC influence on TM displacement (not in transfer functions) and on pressure gain produced by the EC [1], the main conclusion reported about the role of the middle ear cavities was that when cavities were opened the TM displacement was increased by a factor of two at low frequencies. The main restriction of the model presented by Koike et al. comes from the air model. The air was modeled as an elastic solid (as all other structures) with a very low Young modulus. There is a FEM paper [2] that analyzes the effect of mastoid cavity in EC pressures and umbo displacement (UD) (not in transfer function). The authors concluded that the pressure and UDs were slightly influenced by the status of the aditus (open or close). The aditus is a small connection between the TC and the mastoid cavities. Gan et al. have developed a relative complete human AS FEM [3–6], but only one of them [3] focused on the possible mechano-acoustic relation between EC, TM and TC, they presented in their study the influence of eardrum perforations would produce on the EC pressure. In other mammals like cats, there is FEM work presenting a model in which all cavities are modeled, and it is clearly visible and demonstrated that the coupling of the cavities of the middle ear to the eardrum causes a resonance around 5 kHz [7].

Regarding experimental researches, there is evidence that changing middle ear cavities produce changes in middle ear impedances [8], so it should affect the outer and middle ear transfer functions. Other works only shows results in a frequency range up to 4000 Hz [9]. Anyway, these experimental results are not entirely comparable with the effect that the absence of the TC simulated in our FEM would have, obviously opening the TC is a variation in the conditions of the AS, but the cavity remains producing reflecting waves, the air remains and presents some resistance and/or resonances to eardrum deformation. Two experimental paper established a relation between the middle ear cavities and a second resonance in gerbils [10, 11]. In addition, another paper [12] establishes a relation between the vibrations of gerbil TM and the opening of the middle ear cavity.

This paper is based on outer and middle ear numerical simulations developed by means of FEM. The maximum number of possible combinations of different outer and middle ear subsystems have been modeled and simulated: EEC, TM, OC, TC and simplified cochlea (SC). Four main different combinations have been modeled: EEC only attached to TM; EEC coupled to TM and TC; EEC coupled to TM, OC, and SC; and the full model: EEC coupled to TM, TC, OC and SC. Other three combinations have been simulated on the basis on the full model: The TC was opened to an air-filled domain with open boundary conditions that do neither reflect nor dampen the outgoing waves. These situations try to simulate experimental setups at the laboratory tests.

The model presents a complete fluid–structure interaction among EC, TM, TC and oval window. The TM modeling presents a crucial innovation respect to previous models; the elements used in the TM have an improved formulation, the formulation enhanced strain [13], which eliminates the problems of “shear locking” of the elements used in thin membranes. This fact together with a proper mesh convergence analysis provide sufficient and necessary guarantees of correct results in middle ear transfer functions. The results shown in this work are the EC and middle ear transfer functions as follows: EC, the ratio of pressure along the EC and TC to that of the EC entrance (pressure gain); middle ear: the ratio of umbo displacement to tympanic membrane pressure (UD/TMP); the ratio of Umbo velocity to tympanic membrane pressure (UV/TMP); the ratio of stapes displacement to tympanic membrane pressure (SD/TMP); and the ratio of stapes velocity to tympanic membrane pressure (SV/TMP).

Before continuing with the following section of the paper it must be stated what our numerical model does and what it does not. Which results are useful and which can only be considered qualitative.

Apart from geometry uncertainties dues to natural variability, the maximum level of inaccuracy is due to the difficulty to obtain the mechanical properties of some components (TM, tensors, joints…). In this paper, these values have been obtained from bibliography and has been assumed. There is no attempt to discuss its accuracy. In previous works [14], they have been object of a sensitivity analysis in order to establish its influence over the final results. Basically there are two different effect that we can group in two main features, those influencing the stiffness of the system (basically Young modulus) and those that alter the damping (viscoelasticity, damping, acoustic absorption). Even when they have been carefully chosen according to literature, as will be stated in the next section, their potential inaccuracy does not present a great influence and do not affect the main finding of this study.

Despite this, there are two key aspects in the numerical model that must be remarked due to its strong influence on the mechanisms studied. The first one is the proper fluid–structure interaction modeling. Even when there could be some delay effect (phase differences) due to some material viscoelasticity, it is negligible compared with the reflection effect of the cavities and the TM. So this coupled effect must be correctly simulated.

The second one it is related with the difficulty of modeling and meshing the membrane. A proper structure interaction with the fluid at both sides of the membrane requires the use of solid elements. It is a task that can introduce important errors in the model, depending on the mesh size and the finite element formulation.

## Models and methods

Numerical simulations have been performed by means of the FEM using the commercial software ANSYS 13. All numerical simulations consist of harmonic analysis in a frequency range from 100 to 20,000 Hz. All models use a unit value pressure at the entrance of external auditory canals an input signal.

### Geometry of the different parts of the human AS

The geometries of the different subsystems have been obtained from the bibliography. Differences in the geometric and material properties should affect the transfer functions. A previous work [14] presented sensitivity analysis of geometry, providing that if the geometrical measurements fall within a normal range, the transfer function would not be significantly affected. Since all the geometries were obtained from sane ears, the model presented in this paper is representative of the reality.

**Fig. 1:**
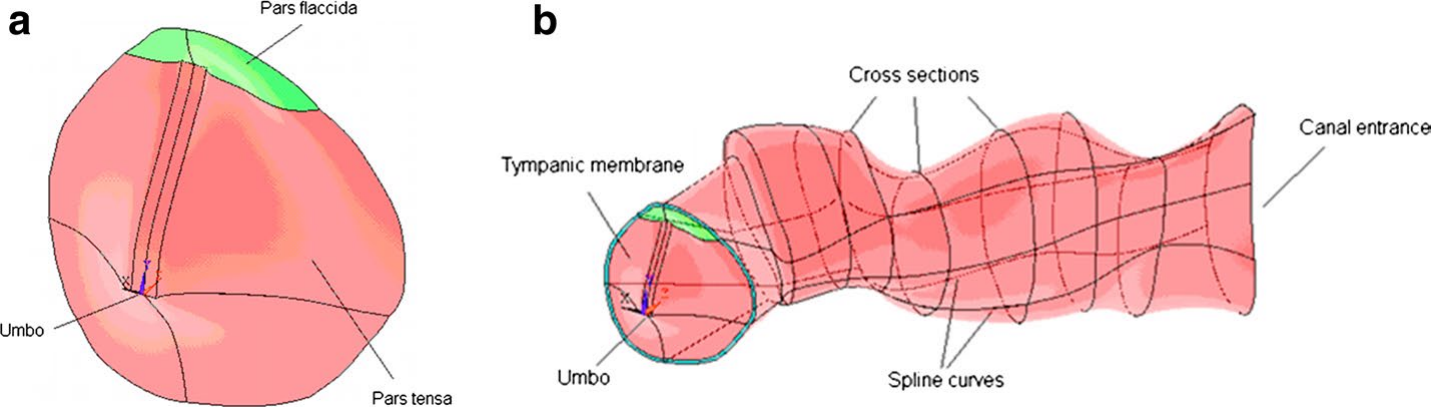
Geometrical model of the tympanic membrane and external canal. **a** Surface of the tympanic membrane **b** volume of the external auditory canal

The geometric model of the TM was obtained from the surface eardrum data, using moiré interferometry [15], with the help of CAD tools. A cloud of points were used to make the surface shown in Fig. 1a.

The auditory canal was obtained with data from nine cross sections obtained from the work published by Egolf [16]. With the help of CAD tools, the contour lines of the sections were generated and then were joined by spline curves. The volume of the canal is made with 69 areas which compose the outer surface as shown in Fig. 1b.

The anatomic measures and functional properties of middle ear were based on published geometric data of human normal middle ear elements. The geometrical model is divided into three parts: the TM or eardrum, the OC (malleus, incus and stapes), and the system of ligaments, tendons and joints that include superior, lateral and anterior mallear ligament, incudal ligament, tensor tympani tendon, stapedial tendon, tympanic annular ring, stapedial annular ligament, incudomallear joint and incudostapedial joint. To achieve the model of each part, a different methodology is used due the differences in physiology, anatomy and mechanical behaviour.

**Fig. 2:**
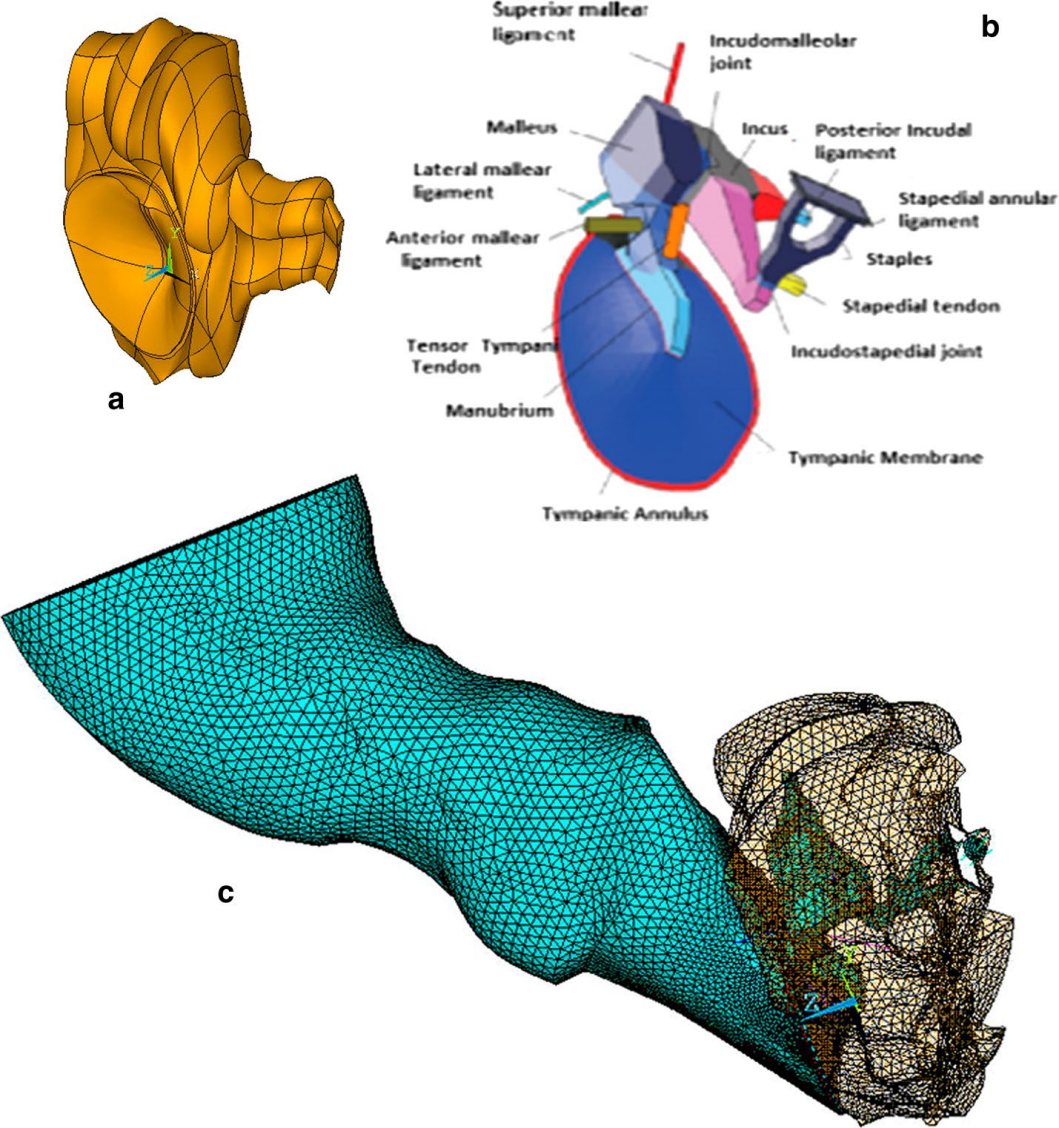
Middle ear and tympanic cavity models. Complete finite element model (FEM) **a** surface of the tympanic cavity **b** 3D geometrical model of the ossicular chain of a human ear. **c** Complete finite element model (FEM)

The OC was drawn using three-dimensional CAD tools from the orthogonal views obtained by Weistenhöfer and Hudde [17] as shown in Fig. 2b. The superior, lateral and anterior mallear ligament and tensor tympani tendon are considered circular section bars according means values [18]. The dimension and shape of stapedial tendon was taken from Cheng and Gan [19]. The incudal ligament was assumed as a square section bar and 0.3 × 0.4 × 0.8 mm [6].

The TC geometry is based on published photomicrographs [20]. Reconstruction of the 3D model was made from 16 sections obtained from the photomicrographs and the result is shown in Fig. 2a.

The geometry of the cochlea has not been modeled. It has been used as an equivalent system consisting of damper-mass-damper inspired in the literature [19], and provides validated results at low computational cost [14]. The complete finite element model is shown in Fig. 2c.

### Elements and properties used in modeling

Fluid: the EEC and TC are modeled emulating the air. The element used is the Fluid 30 of ANSYS 13.0 [13]. The tetrahedral shape is used and the mesh size used is 0.5E−3 m. In all numerical examples the following properties of the fluid are constant: density: 1000 kg/m^3^. Speed of sound: 343 m/s. The acoustic absorption coefficient for the TM and canal wall are 0.007 and 0.02 respectively [3]. The element Fluid 130 has been used to emulated the open boundary conditions of the open TC.

Eardrum and annular ligament of the eardrum: The TM and the annular ligament tympani have been modeled with hexahedral solid element 185 of ANSYS 13. The TM must be meshed as solid elements in order to do a proper fluid–structure connection among EEC, TM and TC. The element uses an improved formulation (formulation enhanced strain) which eliminates the problems of “shear locking” of the elements used in thin membranes. The mesh size used was 200 μm.

**Fig. 3:**
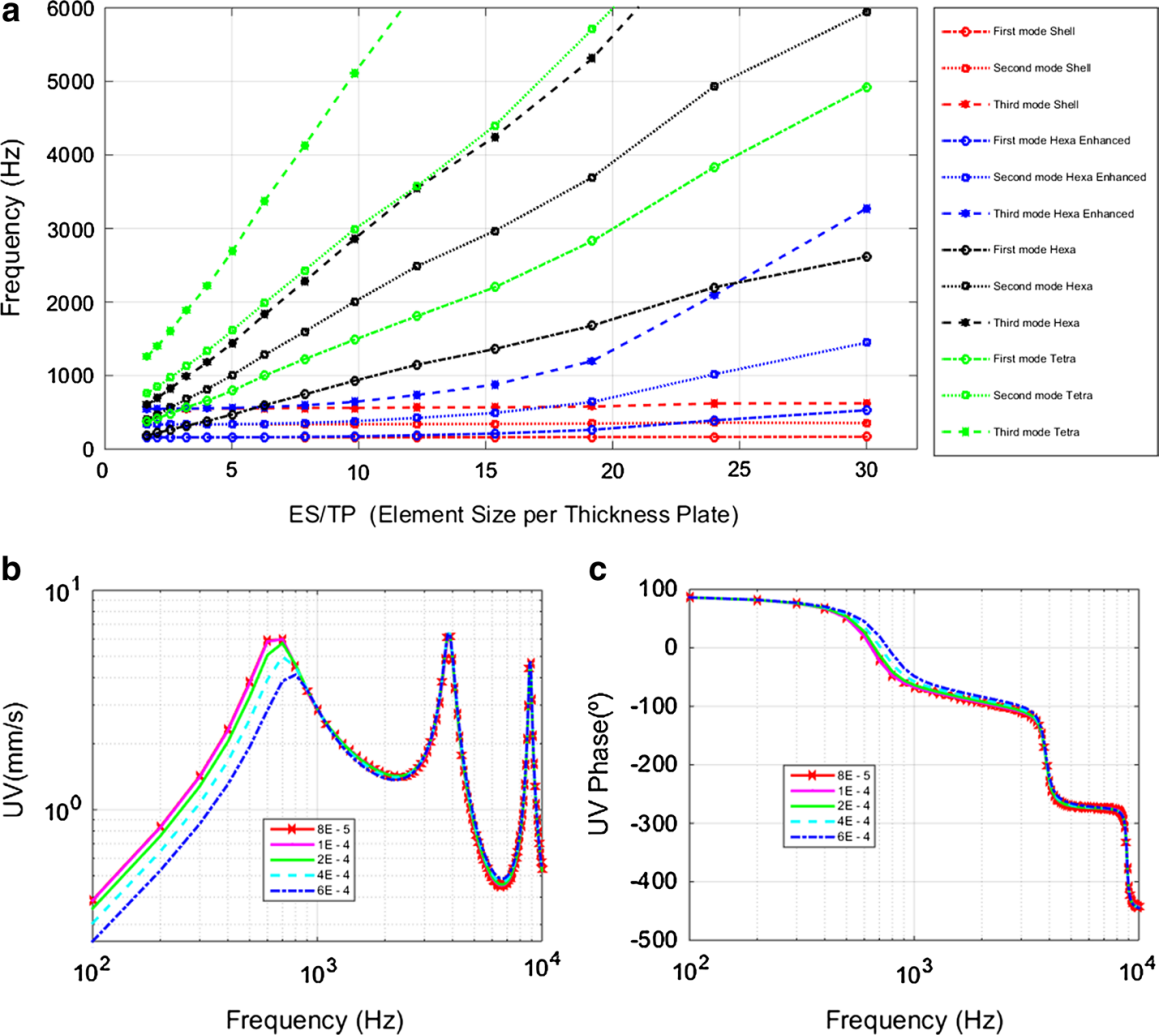
Convergence analysis of element type and integration formulation **a** first three natural frequencies for shell, one hexahedral layer with the formulation enhanced strain, one hexahedral layer and free mesh with tetrahedral elements. Convergence analysis of tympanic membrane size elements. **b** Umbo velocity (UV). **c** Umbo velocity phase (UV phase). For both the size elements analyzed range is from 6E−4 to 8E−5 m

Figure 3a shows a convergence analysis based on a modal analysis applied to an embedded circular plate with a diameter of 1 cm and 50 µm thickness. The figure represents the third first natural frequencies against the dimensionless ratio element size per thickness plate (ES/TP). It has been tested for four different kinds of elements: shell, one hexahedral layer with the enhanced strain formulation, one hexahedral layer and free mesh with tetrahedral elements. Shell results are the target, hexahedral and tetrahedral usually need a lot of elements to reach the objective, but with the new formulation combined with hexahedral shape, the convergence is reached with a ES/TP ratio of 10. This aspect is very critical because of the topology of the membrane with a thickness of 70 μm. The validity of this approach has been tested with an experimental–numerical study [21].

In addition, some mesh convergence analyses were performed to confirm the optimal mesh size. A convergence analysis of TM size elements is shown in Fig. 3b for the UV and in Fig. 2c for the UV phase. The size elements analyzed range is from 6E−4 to 8E−5 m. The optimal mesh size was fixed on 200 μm.

The damping coefficient β was 1 × 10^−4^ s for all solid components. The mechanical properties of the membrane are different for the pars tensa and pars flaccida. Table 1 shows the most important characteristics.

**Table 1:** Mechanical properties used in middle ear components for FEM

Component	Density (kg/m^3^)	Young’s modulus (N/m^2^)	Poisson’s ratio
Eardrum			
Pars tensa	1.2 × 10^3a^	3.2 × 10^7i^	0.3^d^
Pars flaccida	1.2 × 10^3a^	1 × 10^7c^	0.3^d^
Malleus	1.9 × 10^3b^	1.41 × 10^10e^	0.3^d^
Incus	1.9 × 10^3b^	1.41 × 10^10e^	0.3^d^
Stapes	1.9 × 10^3b^	1.41 × 10^10e^	0.3^d^
Tympanic annulus	1.2 × 10^3(supposed)^	6 × 10^5d^	0.3^d^
Manubrium	1.0 × 10^3c^	4.7 × 10^9c^	0.3^d^
Tensor tympanic tendon	2.5 × 10^3c^	2.6 × 10^6c^	0.3^d^
Lateral mallear ligament	2.5 × 10^3c^	6.7 × 10^4d^	0.3^d^
Anterior mallear ligament	2.5 × 10^3c^	2.1 × 10^6d^	0.3^d^
Superior mallear ligament	2.5 × 10^3c^	4.9 × 10^4d^	0.3^d^
Incudal ligament	2.5 × 10^3c^	6.5 × 10^5h^	0.3^d^
Stapedial tendon	2.5 × 10^3c^	5.2 × 10^5c^	0.3^d^
Stapedial annular ligament	2.5 × 10^3c^	2 × 10^5f^	0.3^d^
Incudomallear joint	3.2 × 10^3d^	1.41 × 10^10d^	0.3^d^
Incudostapedial joint	1.2 × 10^3d^	6 × 10^5g^	0.3^d^

^a^Williamns and Lesser [33]

^b^Weistenhöfer and Hudde [17]

^c^Koike et al. [1]

^d^Sun et al. [34]

^e^Speirs et al. [35]

^f^Gan et al. [5]

^g^Prendergast et al. [36]

^h^Gan et al. [6]

^i^Wada and Kobayashi [37]

OC: the malleus, incus, and stapes have been modeled with the Solid45 element in its tetrahedral shape. The mesh size used is 400 μm. The same elements and mesh size have been used to model incudostapedial and incudomallear joints, incudal ligament, and stapedial tendon. The tensor tympani tendon and posterior, anterior and superior ligaments are modeled as linear elements with the Beam4 element (six degrees of freedom at each node). The stapedial annular ligament is assumed to be an elastic band around the footplate 0.1 mm wide and 0.1 mm thick using Shell43 elements. Table 1 shows the most relevant properties of the OC. The damping coefficient β was 1 × 10^−4^ s for all solid components. These property values were subjected to a previous sensitivity analysis [14] in order to validate their values applied to this FEM.

The cochlea is modeled as an equivalent load [6], consisting of a block of rigid mass of 25.5 mg positioned between two groups of 5 dampers to each one. Each group added 0.1 N s/m. They are distributed evenly on opposite sides of the mass block and connected to the center of the footplate. Solid45 elements with infinite stiffness are used for the mass block. Combin14 elements are used for dampers.

The boundary conditions of the model include the suspensory ligaments and tendons of the OC, which is joined at one end with the nodes at the intersection of each bone and at the other end is fixed to simulate the union with the middle ear cavity. The nodes of the outer edge of the ‘tympanic annular ring as well as nodes of the periphery of stapedius annular ligament are fixed to simulate the connection with the middle ear cavity.

### Combinations of finite element models

Seven different combinations have been simulated in order to discern what the impact of each subsystem in the human AS is. In Table 2 is shown a summary of all combinations. In Fig. 2c the full model FE and the OC model with its ligaments and tendons are shown.

**Table 2:**
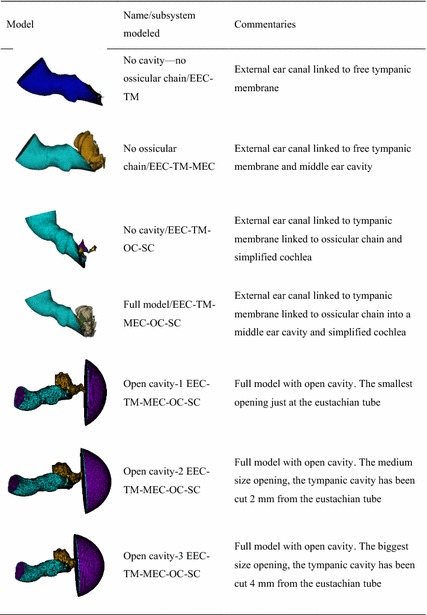
Combinations of FE models simulated by means of FEM

## Results

The results show the TMP to umbo and stapes footplate displacement and velocity transfer function. The comparative of FEM results and experimental results from literature are shown first in order to validate the proposed model. The experimental results are divided in open-cavities (OC) and close-cavities (CC).

### Transfer functions of the eardrum

#### UD/TMP transfer function

**Fig. 4:**
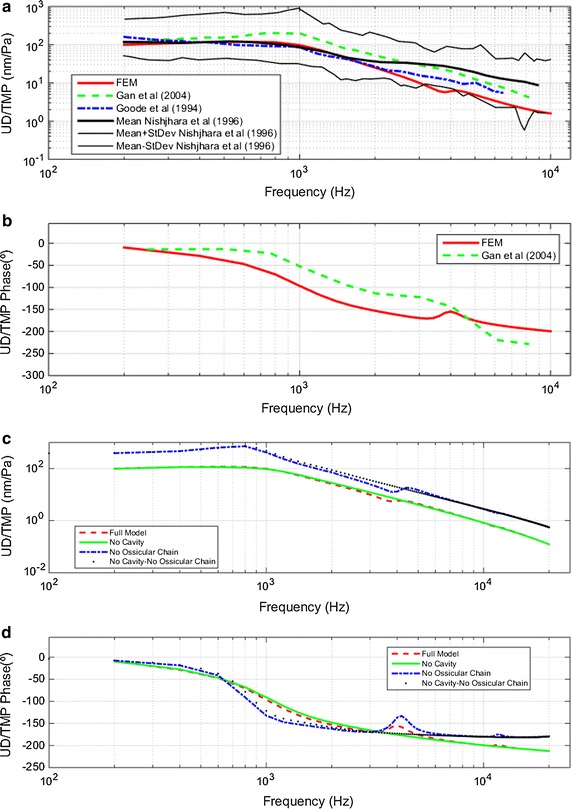
Umbo displacement relative to tympanic membrane pressure (UD/TMP) **a** magnitude comparison of full model-derived umbo velocity vs. sound pressure in the ear canal with published data by Gan et al. [23]-OC, Goode et al. [24]-CC and Nishihara et al. [22]-CC **b** phase angle comparison **c** magnitude results for four different combinations of FEM simulations: **d** phase angle for same four FEM combinations

Figure 4 shows the UD relative to TMP transfer function, UD/TMP. Figure 4a shows a comparison of experimental results with those obtained by the FEM (full model). The correlation between experimental and numerical results is accepted since FEM results are still between the standard deviations reported by Nishihara [22]. Therefore, it is observed in shape and in the peak frequency of response, around 700–1000 Hz. The main difference between experimental and numerical results is above 2000 Hz, where FEM predicts a lower response to the experimental results. There is also a discrepancy in the phase, where FEM decays faster at low frequencies, and at high frequencies it is smoother than experimental results.

Figure 4c, d show the results obtained for UD/TMP magnitude and phase, respectively, for four different combinations of FEM simulations. The main differences between the full model and no cavity model are the two resonance peaks around 4000 and 12,000 Hz that are introduced by the TC attachment. This change is also prominent at the phase of Fig. 4d. The difference between the no cavity–no OC model and the no OC model is the presence of the TC, and the difference between them is the same as in the first two graphs.

It is evident that both the presence of the TC and the OC and cochlea pairing have an effect on the response, but it should be noted that the shape of the responses is similar in all cases. Therefore the TM is undoubtedly the most influential sub-system in the UD/TMP transfer function. A second resonance is only observed when the TC is modeled.

#### UV/TMP transfer function

**Fig. 5:**
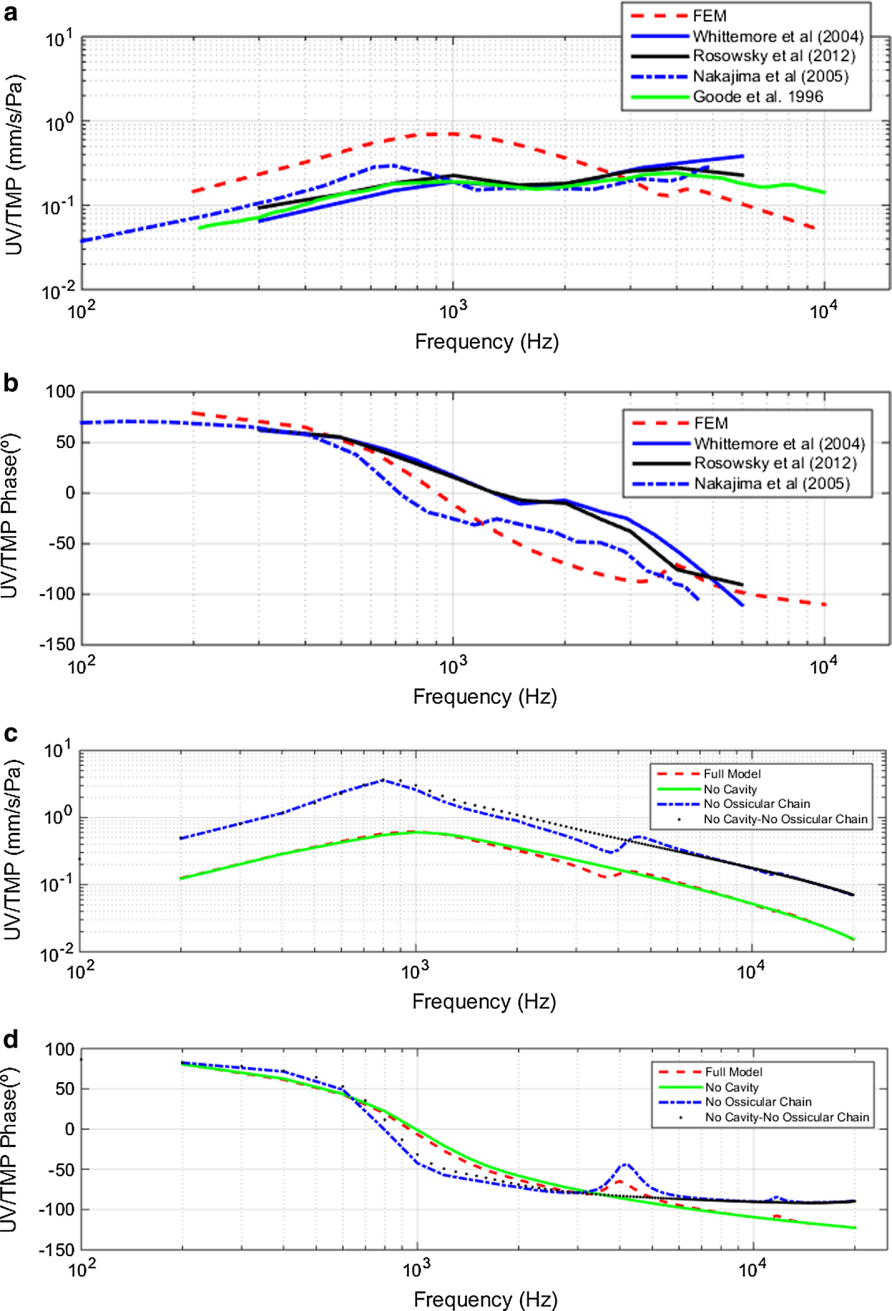
Umbo velocity relative to tympanic membrane pressure (UV/TMP). **a** Magnitude comparison of full model–derived umbo velocity vs. sound pressure in the ear canal with published data by Whittemore et al. [27]-CC, Rosowsky et al. [25]-CC, Nakajima et al. [26]-OC and Goode et al. [24]-CC **b** phase angle comparison **c** magnitude results for four different combinations of FEM simulations **d** phase angle for same four FEM combinations

Figure 5 shows the UV with respect to the EC sound pressure transfer function, UV/TMP. Figure 5a shows a comparison of experimental results with those obtained by the FEM (full model). The results reported by Rosowsky [25] were performed in live humans in contrast with cadaveric results reported by Nakajima [26]. The first resonance in FEM is around 1000 Hz with a value of 0.6 mm/s/Pa. Experimental results show the first resonance at 700 Hz and 0.3 mm/s/Pa [26], and 1000 Hz and 0.2 mm/s/Pa [25]. The second resonance is around 4000 Hz for all results, with higher values for the experimental results.

Figure 5c, d show the results obtained for UV/TMP magnitude and phase for four different combinations of the FEM. The responses of UV/TMP has an identical behavior to those of UD/TMP. A second resonance is only observed when the TC is modeled.

Figures 4c, d and 5c, d show two main effects of the whole OC-cochlea system on the transfer functions: (1), the responses of UV/TMP and UD/TMP are reduced. This reduction was expected and should be proportional to the energy transmitted from the eardrum through the ossicles to the cochlea. And (2), the first resonance frequency of the system changes from 700 to 1000 Hz when the OC and cochlea are modeled. This fact is also observed in the UV/TMP phase, when UV and TMP are in phase. The system is in resonance when the eardrum absorbs more energy from pressure waves coming through the EC. In Fig. 5d, it is shown that in the two models without the OC, the velocity and pressure are in phase at a lower frequency than in models in which the OC is modeled. This is consistent with the idea that the coupling of the eardrum to the OC and cochlea increases the stiffness and damping of the system.

### Transfer functions of the stapes

#### SD/TMP transfer functions

**Fig. 6:**
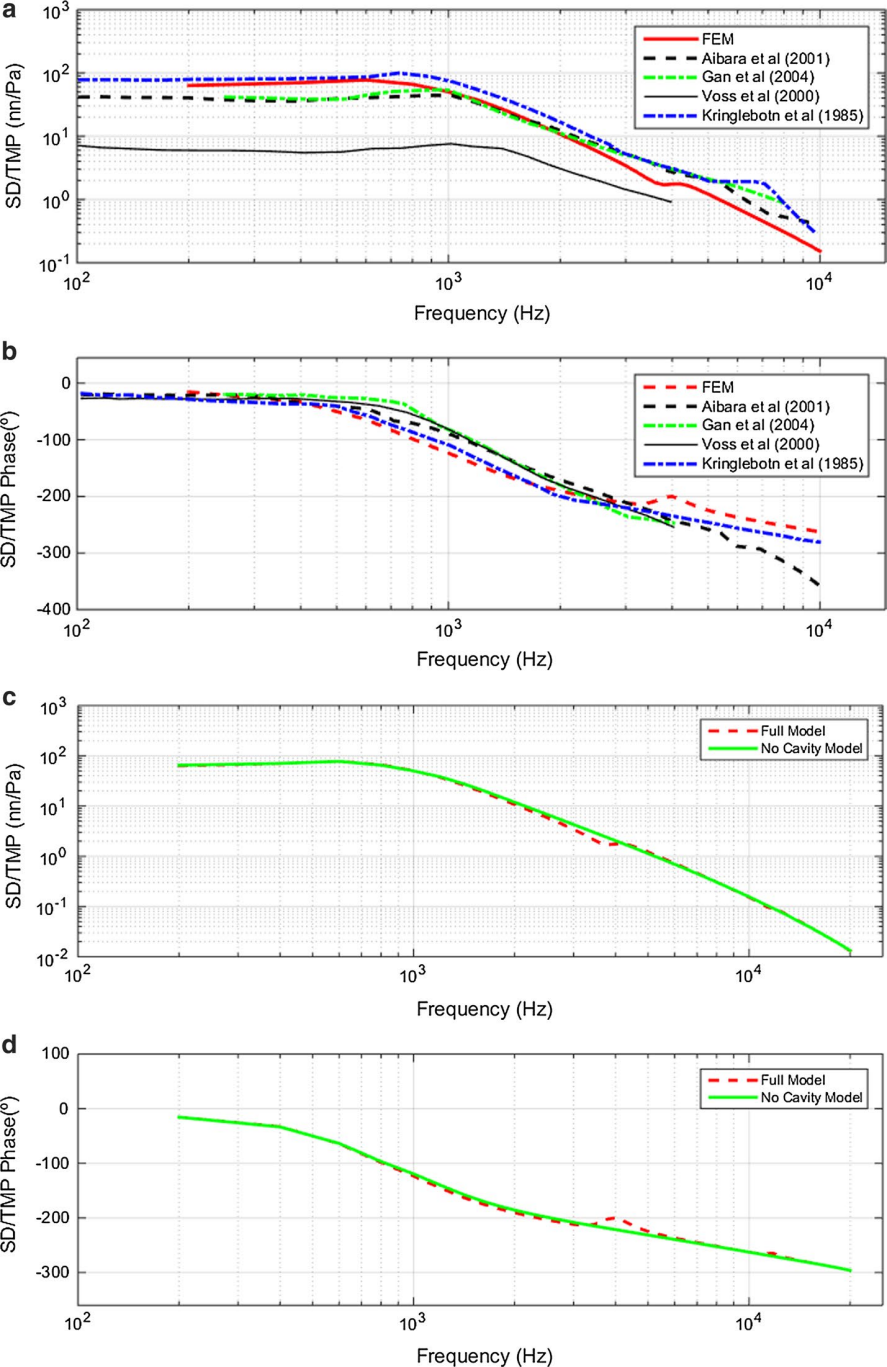
Stapes displacement relative to tympanic membrane pressure (SD/TMP) **a** magnitude comparison of full model–derived Stapes displacement vs. sound pressure in the ear canal with published data by Aibara et al. [28]-CC, Gan et al. [23]-OC, Voss et al. [9]-CC and Kringlebotn et al. [29]-CC **b** phase angle comparison **c** magnitude results for four different combinations of FEM simulations **d** phase angle for same four FEM combinations

Figure 6 shows the SD with respect to the EC sound pressure transfer function, SD/TMP. Figure 6a shows a comparison of experimental results with those obtained by the FEM (full model). We observe a good correlation between experimental and numerical results. The main difference between the experimental and numerical results is the peak frequency being a little lower in the FEM. This is best seen in the phase representation in Fig. 6b, where the FEM phase is slightly ahead of the experimental results. The presence of a second resonance in SD/TMP is noteworthy. In the FEM, this peak is simulated around 4000 Hz. In the experimental results, Aibara’s results are around 5000 Hz [28], and Kringblebotn’s results are around 6000–7000 Hz [29]. The second resonance is not present in the other experimental results.

Figure 6c, d show the results obtained for the SD-TMP magnitude and phase respectively for two different FE models. The main differences between the full model and no cavity model are the response peaks around 4000 and 12,000 Hz. This increase shows up very well in Fig. 6d. These results are consistent with those obtained for the eardrum transfer function.

#### SV/TMP transfer functions

**Fig. 7:**
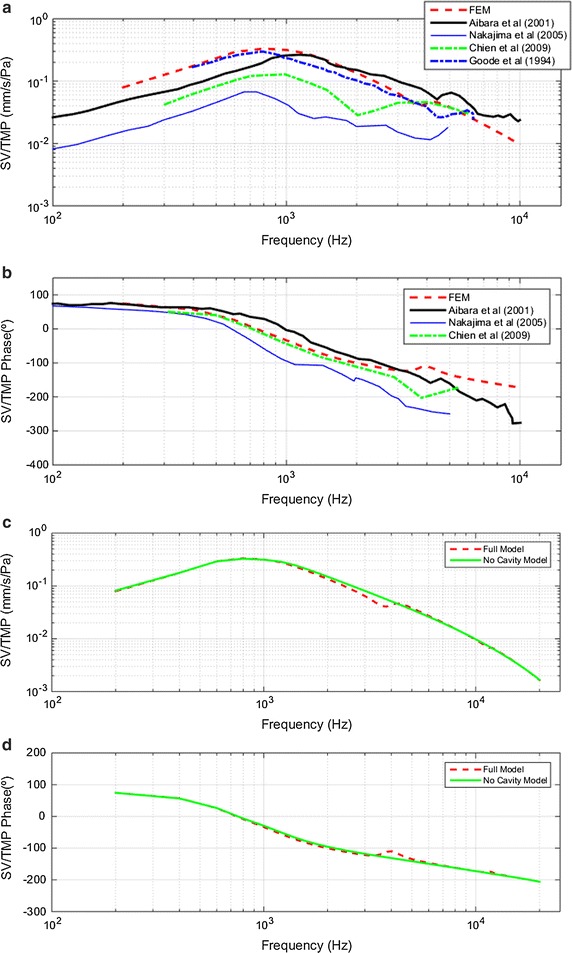
Stapes velocity relative to tympanic membrane pressure (SV/TMP) **a** magnitude comparison of full model-derived stapes velocity vs. sound pressure in the ear canal with published data by Aibara et al. [28]-CC, Nakajima et al. [26]-OC, Chien et al. [30]-OC and Goode et al. [24]-CC **b** phase angle comparison **c** Magnitude results for four different combinations of FEM simulations **d** phase angle for same four FEM combinations

Figure 7 shows the SV with respect to EC sound pressure transfer function, SV/TMP. Figure 7a, b show a comparison of experimental results with those obtained by the FEM (full model). The correlation among numerical and experimental result is acceptable. The first resonance frequency in the FEM is around 800 Hz with a value of 0.32 mm/s/Pa. Experimental results are variable: 0.07 mm/s/Pa at 700 Hz [26], 0.28 mm/s/Pa at 1200 Hz [28], 0.3 mm/s/Pa at 700 Hz [24], and 0.13 mm/s/Pa at 800 Hz [27]. Regarding the second resonance, only two experimental results were performed up to the frequency where this resonance was beginning [24, 26]. The results of Chien [30], et al. demonstrate this second resonance (between 2000 and 6000 Hz) is in a wider frequency range, also the magnitude is variable. FEM simulation fits well with Aibara’s results [28]. Regarding phase representation, there is an adequate correlation except for high frequencies, where FEM reports a phase lower than the experimental ones.

Figure 7c, d show the results obtained for the SV-TMP magnitude and phase respectively for two different combinations of FEM. The interpretation of results is quite similar for SD/TMP.

### Open tympanic cavity

In this subsection, the effect of the gradual TC opening is presented. The TC is connected to an air-filled domain with open boundary conditions that do neither reflect nor dampen the outgoing waves.

The results of three open cavities FEM are shown in this subsection together to the full model FEM. There are many possibilities to consider cavity apertures depending on the experiment setup. For this study, the opening is situated in the Eustachian tube side and different sizes have been analyzed. The open cavity-1 model has the smallest opening just at the Eustachian tube. The open cavity-2 model has a medium size opening, the TC has been cut 2 mm from the Eustachian tube. The open cavity-3 model has the biggest size opening, the TC has been cut 4 mm from the eustachian tube.

**Fig. 8:**
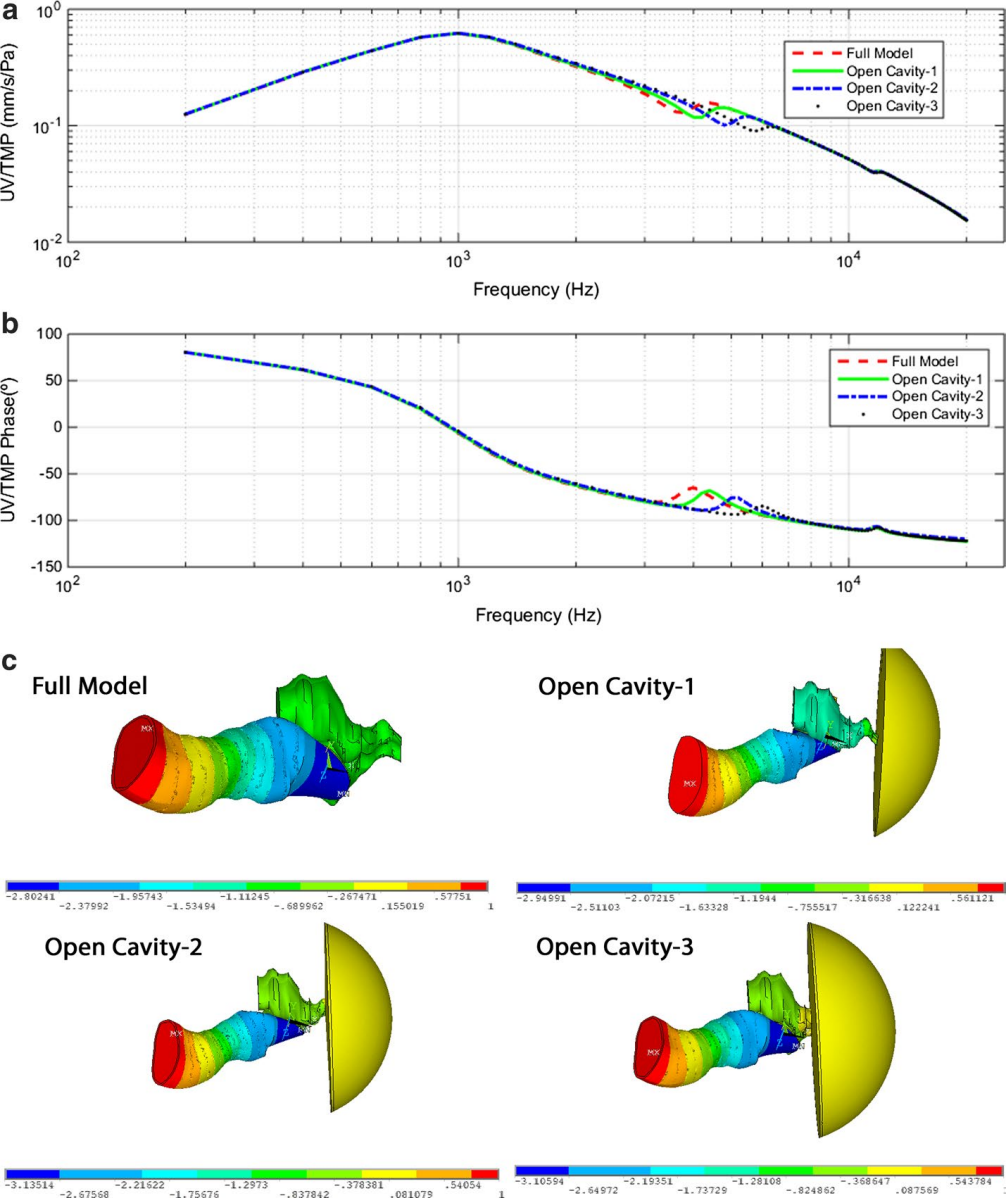
Gradual opening cavity effect on the umbo velocity relative to tympanic membrane pressure (UV/TMP) and pressure distribution. **a** Magnitude results for four different combinations of FEM simulations: full model and open cavities models **b** phase angle for same four FEM combinations. **c** Pressure distribution along external ear canal, tympanic cavity and air-filled cavity at 4200 Hz for same FEM combinations

Figure 8a, b shows the UV with respect to the EC sound pressure transfer function (UV/TMP) for the four FEM combinations. As it was expected, the smaller the opening is, the similar the response is to the full model one. As the opening is bigger, the second resonance tends to be smoother and at higher frequencies. On the other hand, the first resonance has not been affected by the opening.

Figure 8c shows the pressure distribution along EEC, TC and air-filled cavity at 4200 Hz for the full model and the three open cavity models. The TC pressure distribution is affected by the opening and its size.

## Discussion

The results of Figs. 4, 5, 6 and 7 show that all transfer functions shapes are similar to each other. The response begins to rise and reaches a maximum around 700–1000 Hz depending on the displacement and velocity, or eardrum and stapes. From the maximum, response decreases except slight spikes in the frequency ranges around 4000–6000 and 11,000–13,000 Hz. This similarity in the eardrum and stapes transfer function shapes leads us to deduce that the most influential subsystem in these transfer functions is the eardrum.

As described in the “Results” section, the main difference observed between the results in the models with and without TC is the appearance of a second resonance in the transfer functions of the eardrum and stapes. This second resonance is between 3500 and 5000 Hz.

There are differences in the results of eardrum and stapes transfer functions presented in experimental papers. This is justifiable because there are differences between operational methods in the preparation of the temporal bones, the measurement instruments used, and the inherent geometry of each analyzed AS [27]. If we focus on the second resonance at the transfer functions in frequencies from 3000 to 6000 Hz, in some experimental results this second resonance is very noticeable, whereas in some of them it is barely noticeable, and in others is not appreciated. This may be related to the degree of opening of the middle ear cavities shown by the experimental results with measurements made with closed and open cavities [31] (Fig. V7).

When there are more open cavities the second resonance is less noticeable. Kringlebotn results (closed cavities) [29] show the second resonance around 7000 Hz; in the case of Rosowsky results (closed cavities Fig. 9) [25] this recovery is situated between 3000 and 4000 Hz; for Chien results (Fig. 9 open cavities) [30], the recovery of the stapes response is located around 5000 Hz; In Aibara results (closed cavities, Fig. 7) [28] the resonance is around the 5000 Hz; in Goode results (closed cavities) [24] around 6000 Hz; Nakajima (open cavities, Figs. 2, 3, 4 and 5) [26] represents the transfer function from EC pressures to scale vestibule pressures. These results are extrapolated to SV/TMP [29] and a second resonance is also seen in the response around 8000 Hz. The difference in the frequency of the second resonance could be due to geometrical differences and boundary conditions in the middle ear cavities, as action protocols differ in the temporal bone.

Volandri (Fig. 5) [32] presents a compilation of results from displacement of the eardrum in humans showing many Tympanic displacement results. The second resonance is seen in some articles and not in others. The second resonance is clearly seen in the experimental results for which the middle ear cavities were closed. The experimental results where the second resonance did not appear, were obtained with open Cavities [26]. In other results where the second resonance appears, the middle ear cavities were also open [26, 30].

Therefore, there is a controversy on this fact. There are at least two considerations to be taken into account for explaining this issue: most of experimental results show the mean of several measurements in different individuals and the second resonance frequency can be different due to geometrical and boundary conditions differences of the middle ear cavities. This fact could lead to a cancelation for the resonance. Another important fact is that opening the middle ear cavity does not imply the elimination of the cavities (this FEM paper models closed cavity or nonexistence of cavity), but it implies a change in boundary conditions. The middle ear cavity remains with different conditions and most of the middle ear walls remain reflecting waves. Evidently, the resonance frequencies will change.

This second resonance in transfer functions is also observed in other mammals. Chien et al. [30] shows a compilation of SV/TMP results of different animals such as chinchillas, cats, gerbils, guinea pigs and also live and cadaveric humans. There are authors that justify the relationship between the second (even third) resonance and the presence of middle ear cavities in other mammals, Ravicz [11] justifies this relationship in the coupling of the air masses of the TC and bulla hole in the case of the gerbil.

There are also representative jobs in FE in mammals like the cat. Tuck-Lee [7] presents a model for which all cavities are modeled. Tuck-Lee’s article demonstrates the coupling of the cavities of the middle ear to the eardrum causes a resonance around 5 kHz (Figs. 9 and 11) [7].

The difference between experimental and numerical results above 2 kHz observed in Fig. 4a, may be due to the FEM being modeled with elastic properties rather than viscoelastic, which would fit the real properties of the biological tissues better [4]. There are other works [38, 39] that analyze the differences obtained in pressures and displacement modeling with elastic or viscoelastic properties. Recently De Greef et al. [40] rejected the used of Rayleigh damping on finite element modeling. They supported their conclusion on the delay observed on the experimental results (obtained by means of digital holography). Their conclusion are limited because the finite element model they used to simulate the problem did not included the air surrounding the membrane. Sound pressure was directly applied at the membrane and the influence of the air cavities is missing on their numerical results.

Due to the complexity of these numerical models is difficult to conduct experimental–numerical work to obtain robust conclusion in this matter. Only with simplified and limited experimental setups we can fit the numerical model with the experimental data [21].

In any case, what is relevant in this discussion is that this difference does not affect the specific objectives and conclusions of this research, which is not to assess the effect of mechanical properties of tissues on the response of the AS, but to assess the influence of the auditory subsystem. In this sense, those properties related to the stiffness and mass of the system are very sensitive (as elastic modulus, dimensions, etc.) as they change the dynamics of the system. But those related to energy loss only present a limited influence on the magnitude of the response.

Otherwise, it must be recall that once the mechanical properties have been fixed and justified in our research, FEM results are inside the standard deviations reported by Nishihara [22].

## Conclusions

This study has found evidences resulting that the presence of the TC in the AS introduces a second resonance in middle ear transfer functions at frequencies higher than 3 kHz. It is in coherence with the experimental results even when the experiments have limitations at higher frequencies.

The gradual TC opening affects to the middle ear transfer functions at high frequency. This fact could have a clinical impact, in some common surgical interventions, the size of the TC is modified, deriving on an audition quality lost at high frequencies. On the other hand, the gradual TC opening should be studied deeper to understand interpretation of experimental work which commonly has to manipulate the TC in order to carry out the experiment.

The TM must be carefully meshed when EEC and TC are modeled. Solid element with a determined size (by convergence analysis) and enhanced strain formulation (which eliminates the problems of “shear locking”) must be used to avoid an excess of stiffness.

The eardrum is the most influential subsystem in the Umbo transfer functions shape (UD/TMP, UV/TMP). UD/TM and UV/TMP magnitudes are also strongly affected by the OC and the cochlea attachment, but phase magnitude is only slightly affected. A cascade-like phase distribution is observed in UD/TMP and UV/TMP transfer functions.

## Abbreviations

AS: auditory system
EC: ear canal
EEC: external ear canal
FEM: finite element method
OC: ossicular chain
SC: simplified cochlea
SD: stapes displacement
SV: stapes velocity
TC: tympanic cavity
TM: tympanic membrane
TMP: tympanic membrane pressure
UD: umbo displacement
UV: umbo velocity
